# *map3k1* is required for spatial restriction of progenitor differentiation in planarians

**DOI:** 10.1101/2025.03.04.641450

**Published:** 2025-03-04

**Authors:** Bryanna Isela-Inez Canales, Hunter O. King, Peter W. Reddien

**Affiliations:** 1 Whitehead Institute for Biomedical Research, Cambridge, MA 02142, USA.; 2 Department of Biology, Massachusetts Institute of Technology, Cambridge, MA 02139, USA.; 3 Department of Brain and Cognitive Sciences, Massachusetts Institute of Technology, Cambridge, MA 02139, USA; 4 Howard Hughes Medical Institute, Massachusetts Institute of Technology, Cambridge, MA 02139, USA.

## Abstract

Planarian regeneration and tissue turnover involve fate specification in pluripotent stem cells called neoblasts. Neoblasts select fates through the expression of fate-specific transcription factors (FSTFs), generating specialized neoblasts. Specialized neoblasts are spatially intermingled and can be dispersed broadly, frequently being far from their target tissue. The post-mitotic progeny of neoblasts, serving as progenitors, migrate and differentiate into mature cell types. Pattern formation is thus strongly influenced by the migratory assortment and differentiation of fate-specified progenitors in precise locations, which we refer to as progenitor targeting. This central step of pattern maintenance and formation, however, is poorly understood. Here, we describe a requirement for the conserved *map3k1* gene in targeting, restricting post-mitotic progenitor differentiation to precise locations. RNAi of *map3k1* causes ectopic differentiation of eye progenitors along their migratory path, resulting in dispersed ectopic eyes and eye cells. Other neural tissues similarly display ectopic posterior differentiation and pharynx cells emerge dispersed laterally and anteriorly in *map3k1* RNAi animals. Ectopic differentiated cells are also found within the incorrect organs after *map3k1* RNAi, and ultimately teratomas form. These findings implicate *map3k1* signaling in controlling the positional regulation of progenitor behavior – restricting progenitor differentiation to targeted locations in response to external cues in the local tissue environment.

## Introduction

Planarians are capable of regenerating any missing body part through the action of pluripotent stem cells called neoblasts (Reddien, 2018). Neoblasts also maintain all cell types of the adult body through a process of constitutive cell turnover. Because planarians have >125 different adult cell types, these stem cells must be capable of choosing among a large array of possible cell fates (Fincher et al., 2018; Plass et al., 2018; Zeng et al., 2018; King et al., 2024). Fate specification can occur in neoblasts through the activation of transcription factors called fate-specific transcription factors (FSTFs), producing specialized neoblasts (Reddien, 2022). Specialized neoblasts divide and can produce daughter cells that act as migratory precursors (post-mitotic progenitors) for differentiated cell types (Eisenhoffer et al., 2008; van Wolfswinkel et al., 2014; Reddien, 2022; King et al., 2024).

Fate choice in neoblasts can be regulated by position. For instance, eye-specialized neoblasts are formed in roughly the anterior third of the animal (Lapan and Reddien, 2011, 2012). However, this spatial regulation of stem cell fate specification is coarse, when compared to the precise positioning of cell differentiation to form functional, complex tissue architecture (Lapan and Reddien, 2011, 2012; Adler et al., 2014; Scimone et al., 2014a; van Wolfswinkel et al., 2014; Park et al., 2023). Because specialized neoblasts are produced in broad regions, they are often found far from their target tissue. Indeed, specialized neoblasts can frequently be found closer to other differentiated cell types than to the differentiated tissue they are fated to mature into (Park et al., 2023). Furthermore, neoblasts are specified in a highly spatially intermingled manner, in heterogenous neoblast neighborhoods (Park et al., 2023). For instance, a muscle-specialized neoblast could have a neural, intestinal, epidermal, eye, or other specialized neoblast as its nearest neoblast neighbor (Park et al., 2023). These observations suggest that the regulation of differentiation programs for post-mitotic migratory progenitors is a crucial aspect to patterning and maintaining the various tissues in the animal. Neoblasts themselves are not highly migratory, however (Saló and Baguñà, 1985; Guedelhoefer and Sánchez Alvarado, 2012; Abnave et al., 2017). By contrast, the post-mitotic descendant cells of neoblasts can remain immature for a number of days – serving as progenitors – and can be migratory (Eisenhoffer et al., 2008; Wenemoser and Reddien, 2010; Guedelhoefer and Sánchez Alvarado, 2012; van Wolfswinkel et al., 2014; Abnave et al., 2017; Park et al., 2023). These findings generate a model for pattern maintenance during tissue turnover and formation in planarian regeneration in which specialized neoblasts generate post-mitotic progenitors that assort and target precise locations for differentiation through migration (Park et al., 2023).

From a messy state of planarian progenitor formation, order must arise. Terminal differentiation of post-mitotic progenitors must be regulated to occur at precise positions to prevent disordered differentiation along migratory trails. Defects in this process could lead to errors in tissue pattern, such as supernumerary organs, ectopic differentiation of single cells, incorrect incorporation of a particular cell type into the wrong tissue, and teratoma formation. Therefore, understanding how migratory progenitors know when and where to terminally differentiate into a mature stationary cell is a central problem for understanding how systems of migratory progenitors generate and maintain pattern. Little is known about the molecular regulation of differentiation, restricting it to occur in precise locations. Given that progenitor migratory targeting and differentiation are the predominant processes involved in planarian adult tissue patterning, identification of the molecular processes regulating these behaviors is central for understanding adult regeneration and pattern maintenance. Planarians present the opportunity to uncover general mechanisms underlying migratory progenitor targeting and differentiation regulation, which could apply to a myriad of developmental and regenerative contexts across the animal kingdom.

## Results

### *map3k1* RNAi results in the ectopic posterior differentiation of eye cells along the AP axis.

**Figure 1.**
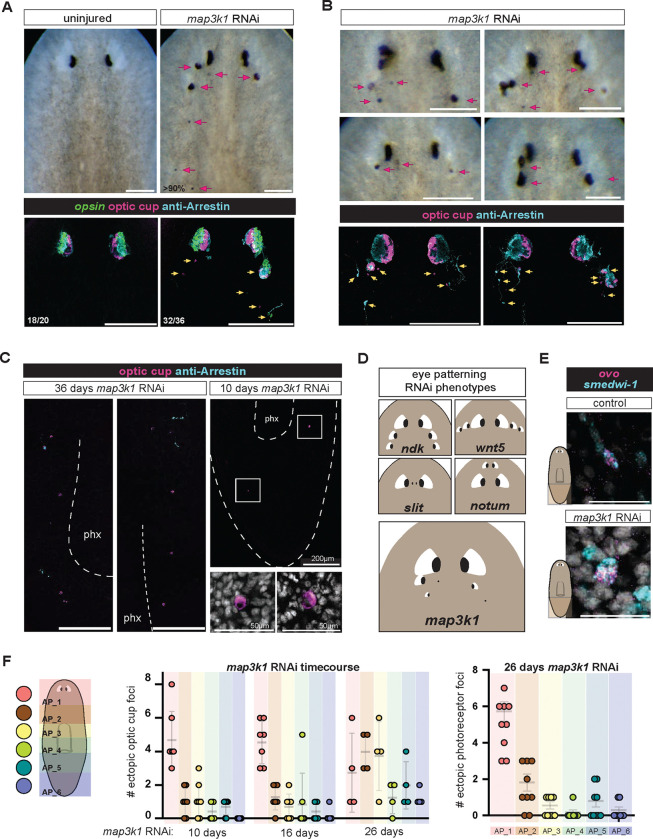
*map3k1* RNAi results in ectopic eyes and ectopic isolated eye cells. **A.** Live images (upper panels) showing posterior eyes in *map3k1* RNAi animals. Upper panels, 6 feedings of RNAi over 36 days. FISH (bottom panels): single optic cup and photoreceptor cells are present ectopically with RNA probes for *opsin* (photoreceptors), a *catalase/tyrosinase/glut3* pool (optic cup), and an anti-Arrestin antibody (labeling photoreceptors and their projections). **B.** Examples showing a diversity of ectopic eyes and eye cells in *map3k1* RNAi animals. **A, B.** Dorsal up; Scale bar, 200μm. **C.** Schematic comparing previously identified eye-patterning phenotypes with the *map3k1* RNAi phenotype. **D.** FISH of single cells differentiated proximal to the pharynx and in the tail of *map3k1* RNAi animals. Dorsal up; scale bar, 100μm. **E.** FISH examples of *ovo*+; *smedwi-1*+ cells in the tail. **F.** Left graph depicts the number of ectopic optic cup differentiation events per animal along the AP axis over 10, 16, and 26 days. Right graph depicts the number of ectopic photoreceptor neurons at 26 days of *map3k1* RNAi; See also Figure 1- figure supplement 1.

We sought to understand how the differentiation of fate-specified progenitors is spatially regulated to control planarian patterning through RNAi studies. The RNAi of a single gene (*map3k1,* dd_5198) encoding a MAP3K1-like signaling protein resulted in a striking phenotype of ectopic eye formation that was distinct in nature from previously described planarian patterning phenotypes, and was suggestive of having a candidate role in progenitor targeting (Figure 1A). We therefore selected this gene for detailed investigation given the robust and unique nature of its patterning phenotype. Planarian *map3k1* encodes a member of the MAP3K1 subclass of MAP3K signaling proteins. MAP3K1 proteins, including the planarian ortholog, possess a unique domain architecture among MAP3Ks. Aside from its characteristic kinase domain, MAP3K1 has other unique domains: a PHD-like RING finger, a SWIM-type RING finger, and a TOG-like domain. These domains have been implicated in non-canonical MAP kinase signaling cascades that enable MAP3K1 to act as a ubiquitin ligase, a scaffold protein, and to signal through MAP kinase proteins (Figure 1- figure supplement 1).

The bilaterally symmetric anterior location of planarian eyes represents the normal targeting location of eye progenitors (Lapan and Reddien, 2011, 2012). RNAi of *map3k1* resulted in the gradual emergence of ectopic eyes posterior to the normal eye location (Figure 1A,B). Ectopic eyes displayed some variance in their posterior and medial-lateral (ML) positions in the pre-pharyngeal animal region (Figure 1A,B). Fluorescent in situ hybridization (FISH) experiments showed that both optic cup and photoreceptor cells were present ectopically (Figure 1A,B). Ectopic optic cup and photoreceptor cells were observed both as clusters of cells that nucleated and grew into additional eyes, as well as individual, isolated eye cells (Figure 1A,B; Figure 1- figure supplement 1B). Isolated, individual photoreceptor neuron or optic cup cell differentiation is rarely observed in the wild-type state. Surprisingly, although ectopic eye cells appeared most frequently in the normal zone of eye progenitor specification, they also appeared along the entire AP axis, including in the posterior half of the tail – far from the normal eye-progenitor specification zone (Figure 1C, Figure 1- figure supplement 1B). Most animals had at least one ectopic eye cell in the tail after 26 days of *map3k1* RNAi. The *map3k1* RNAi phenotype did not appear to result from a anterior-to-posterior progression of eye formation, because isolated eye cells were observed in RNAi animal tails even at early RNAi timepoints when no, or very few other ectopic differentiated eye cells appeared between the head and tail (Figure 1C, F, Figure 1- figure supplement 1B). The very posterior ectopic eye cells that appeared in *map3k1* RNAi animals were more sparse than those in the head, and more likely to appear as singletons rather than aggregates (Figure 1C, Figure 1- figure supplement 1B).

The location where dispersed eye progenitors migrate towards and undergo terminal differentiation is referred to as the target zone (TZ) (Atabay et al., 2018). A variety of genes can be inhibited to result in eye formation in ectopic positions along the anterior-posterior (AP) axis: for instance, posteriorly for RNAi of *nou darake (ndk)* (Cebria et al., 2002) or *wntA* (Kobayashi et al., 2007), and anteriorly for RNAi of *notum* (Hill and Petersen, 2015) and *nr4A* (Li et al., 2019). Furthermore, *wnt5* RNAi causes ectopic lateral eye formation and *slit* RNAi cause medial eye formation (Oderberg et al., 2017; Atabay et al., 2018). However, in all of these cases ectopic eyes appear along particular AP or ML trajectories (Figure 1D, summary cartoon), consistent with alteration of the eye progenitor TZ along a single (AP or ML) axis, but still with differentiation constrained to occur in a particular position on the orthogonal axis. By contrast, *map3k1* RNAi ectopic eyes were more diffuse, often being ectopic in both AP and ML position. This distinct patterning phenotype raised the possibility that *map3k1* was required for a patterning process not previously perturbed by inhibition of prior patterning genes.

Patterning in planarians prominently involves constitutive and regional expression of genes constituting positional information referred to as position control genes (PCGs) (Reddien, 2018). PCGs are predominantly expressed in planarian muscle (Witchley et al., 2013). *slit, wnt5, ndk, nr4A,* and *notum* are regulated in their spatial expression and are components of this PCG patterning system. Therefore, these genes likely influence progenitor-extrinsic cues that guide progenitor targeting. By contrast, *map3k1* is not expressed overtly in a spatially restricted manner, instead being expressed broadly across tissues (Figure 1- figure supplement 1C).

The appearance of eye cells in the tail of *map3k1* RNAi animals suggests that some eye progenitor specification is likely occurring in the tails of these animals. Eye specialized neoblasts can be recognized by the co-expression of the eye-specific FSTF *ovo* (Lapan and Reddien, 2012) and the neoblast marker *smedwi-1* (Reddien et al., 2005). Indeed, *ovo+; smedwi-1*+ neoblasts were observed at low frequency in the midbody and tail of *map3k1* RNAi animals (Figure 1E; Figure 1- figure supplement 1D, E). The frequency of these observed rare *ovo*+ neoblasts was not substantially different from a low frequency observed in control animals, raising the possibility that low frequency sporadic eye neoblast specification events in the body can lead to ectopic differentiation when *map3k1* is inhibited.

### *map3k1* prevents the posteriorization of some but not all anterior cell types

**Figure 2.**
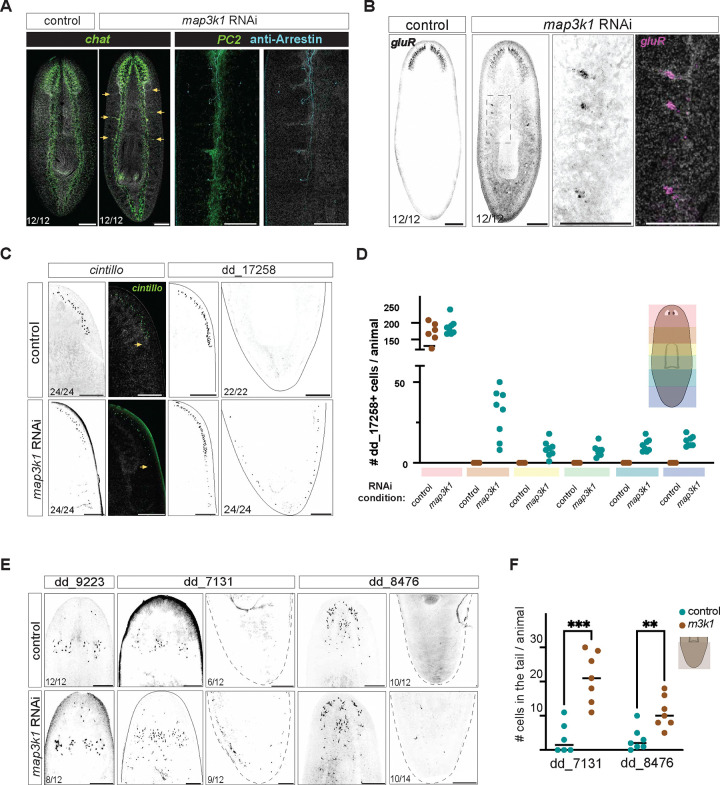
*map3k1* RNAi results in ectopic neuron classes and gland cells along the AP axis. **A.** *map3k1* RNAi animals exhibit posterior ectopic brain branches with *chat*+ neurons and *gluR*+ (dd_16476) neurons. Photoreceptor axons extend along ventral nerve cords and into ectopic brain branches. Ventral up; Scale bars, 200μm; Closeup images, scale bar, 100μm. **B.** FISH images showing no change in *cintillo*+ neuron distribution, but expansion of dd_17258+ cells down the entire AP axis. ventral up; scale bars, 200μm; See also Figure 2- figure supplement 1. **C.** Graph showing distribution of dd_17258+ cells along the AP axis in control and *map3k1* RNAi animals. **D.** Quantification of cells in the tail for dd_7131 and dd_8476. More cells are observed in the tails of *map3k1* RNAi animals compared to control animals; Student t-test using Graphpad Prism software ***p<.0001; **p<.001 . **E.** FISH examples of unaffected (dd_9223) and affected (dd_7131 and dd_8476) parenchymal cell types in *map3k1* RNAi animals. ventral up; scale bar, 200μm. Numbers of animals are represented on the bottom left of each panel.

To assess whether the ectopic differentiation of progenitors that occurred in *map3k1* RNAi animals was specific to the eye, we visualized other features of differentiated tissue patterns. For example, lateral branches are normally associated specifically with the planarian brain (Hyman, 1951). However, after *map3k1* RNAi, ectopic brain branches emerged from the two ventral nerve cords – most commonly observed in the anterior and mid-body, extending to the far posterior of the animal over long-term (>6 weeks) RNAi in uninjured animals (Figure 2A, Figure 2- figure supplement 1A). Ectopic eyes sent axons that traveled along branches and main nerve cord tracts (Figure 2A, Figure 2- figure supplement 1A). Ectopic brain branches that emerged from ventral nerve cords contained *GluR*^+^ neurons, confirming that they are at least partly comprised of normally brain-branch-restricted neurons (Figure 2B). *GluR*^+^ neurons are similarly found in ectopic brain branches observed after RNAi of *ndk* (Cebria et al., 2002).

Another anterior neural population, marked with an RNA probe to dd_17258, also displayed ectopic differentiation across the AP axis of *map3k1* RNAi animals – including through to the tip of the tail (Figure 2C, Figure 2- figure supplement 1B,C). By contrast, the AP location of *cintillo*^+^ neurons, which display posteriorization in *ndk* RNAi animals (Scimone et al., 2016), remained unaffected in *map3k1* RNAi animals (Figure 2C). Another anterior-restricted neural population (*glutamic acid decarboxylase, gad*+) also failed to show posteriorization following *map3k1* RNAi (Figure 2-figure supplement 1D). These findings suggest that *map3k1* is required for the normal AP restriction of a subset of neural cell types during tissue turnover. The cell types that displayed posteriorization following *map3k1* RNAi also maintained their general tissue structure in their normal locations, rather than being lost anteriorly and replaced with a more posterior domain.

To determine if tissue posteriorization occurred in non-neuronal cell types, we assayed particular gland cell populations that predominantly reside in the head, with a small fraction extending down to the pharynx and rarely to the tail. *map3k1* RNAi animals showed posteriorization of gland cell distributions for the primarily anterior dd_7131+ and dd_8476+ populations (Figure 2D–F). As was the case with the neurons, not all gland cell types were affected by *map3k1* RNAi. dd_9223+ cells, which are normally restricted to the anterior, did not change in distribution (Figure 2D). These data indicate that *map3k1* broadly affects the spatial distribution of differentiated cell types for multiple tissues during tissue maintenance through turnover, but that its role in regulating differentiation is restricted to a subset of progenitor populations.

### *map3k1* inhibition causes ectopic anterior differentiation of pharynx progenitors

**Figure 3.**
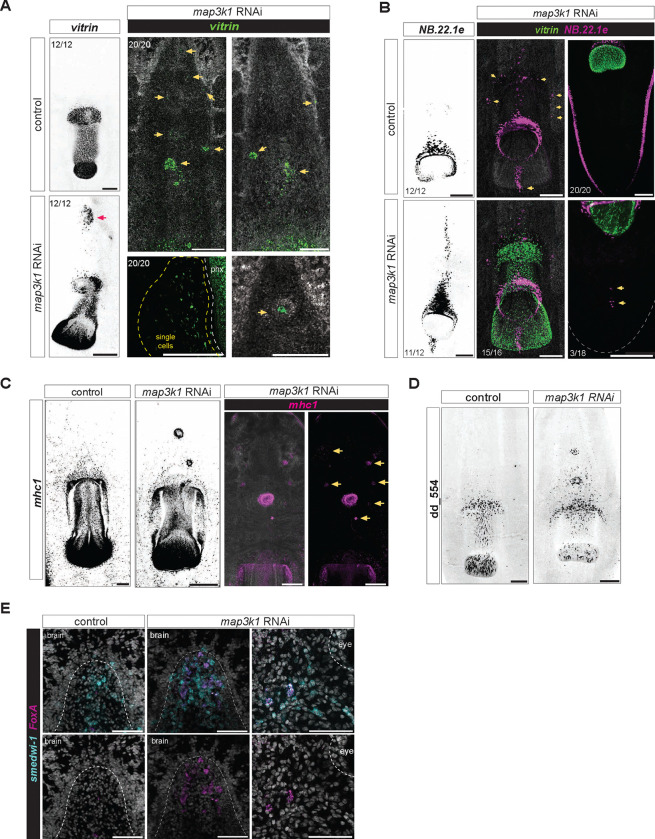
*map3k1* RNAi results in pharynx cell types in ectopic anterior locations. **A.** FISH images showing anterior expansion and disorganization of *vitrin*+ (pharynx-specific marker) cells along the AP-axis. Ventral up; scale bars, 100μm. **B.** FISH showing anterior expansion of *NB.22.1e* (mouth and esophagus) cells along the AP axis. *map3k1* RNAi animals exhibit a low frequency of *NB.22.1e* cells in the tail, posterior to the normal mouth location. **C.** FISH shows anterior expansion of *mhc1*+ cells in *map3k1* RNAi animals compared to control animals. ventral up, scale bar, 100μm. **D.** FISH shows anterior expansion of dd_554 cells (pharynx progenitors) in *map3k1* RNAi animals compared to control animals. Ventral, up. Scale bar, 100μm. **E.** FISH shows *FoxA*+; *smedwi-1*+ cells close to the brain lobes and eyes. Scale bar, 50μm.

Another regional tissue that is maintained through turnover from regional progenitors is the planarian pharynx. *FoxA* is expressed in neoblasts surrounding the centrally located pharynx, specifying progenitors for this organ (Adler et al., 2014; Scimone et al., 2014a). These progenitors must enter the pharynx through a connection to the body at the anterior end of the organ. After *map3k1* RNAi, *vitrin*^+^ pharynx cells appeared at the anterior domain of the predicted *FoxA*+ zone, through which pharynx progenitors are migrating, as well more anterior to the normal *FoxA*+ zone (Figure 3A). *vitrin*^+^ cells clustered together to form small structures containing pharyngeal cells between the original pharynx and the brain (Figure 3A). Most animals developed one main ectopic pharynx cell cluster anterior to the original pharynx, whereas others developed multiple anterior structures that could also exist lateral to the midline (Figure 3A). This variable placement of ectopic pharyngeal cells on the ML axis is reminiscent of the patterning defect observed with eyes described above. Interestingly, many progenitors in the *FoxA* zone appear to differentiate as singletons – single pharynx cells, a phenotype not previously observed with other patterning phenotypes, and also bearing similarity to the ectopic eye cell defect.

The planarian mouth is an epidermal opening associated with the posterior end of the pharynx. Ectopic anterior mouth cells marked by RNA transcripts for the gene *NB.22.1e* were also observed following *map3k1* RNAi. Ectopic mouth cells appeared as an anterior streak of cells with *NB.22.1e* transcripts, originating from the original mouth. In addition, ectopic sparse, scattered mouth cells were also present, sometimes lateral to the midline (Figure 3B). Rarely (n=2/12 animals), an ectopic focus of *NB.22.1e*+ mouth cells was observed in the tail, posterior to the pharynx (Figure 3B). We also examined the pattern of cells expressing *mhc1*, which marks pharyngeal muscle. Similar to the case of *vitrin*+ cells, scattered ectopic foci of pharyngeal muscle cells were observed in the anterior half of the animal (Figure 3C).

Because differentiated pharynx cells were observed outside of the normal progenitor specification zone, we considered the possibility that pharynx progenitor specification itself occurred in ectopic locations following *map3k1* RNAi. dd_554 transcripts mark a post-mitotic pharynx progenitor population (Zhu et al., 2015). Ectopic dd_554 cells were observed anterior to the pharynx in *map3k1* RNAi animals, including in foci (Figure 3D). *FoxA*^+^*; smedwi-1*^+^ cells include prominently pharynx progenitors. A small frequency of animals exhibited *FoxA*^+^*; smedwi-1*^+^ cells between the lobes of the cephalic ganglia of long term *map3k1* RNAi animals. This location is anterior to the normal progenitor specification zone for the pharynx (Figure 3E), which is typically centrally restricted on the AP axis (Adler et al., 2014; Scimone et al., 2014a). These findings are consistent with the interpretation that these *FoxA*^+^*; smedwi-1*^+^ cells possibly include pharynx progenitors and that stem cell fate specification may occur outside of the normally spatially restricted domains in *map3K1* RNAi animals, at least in the case of the pharynx.

Complete posterior duplication of the pharynx has previously been observed with *ptk7, ndl-3*, and *wntP-2* RNAi (Scimone et al., 2016; Hill and Petersen, 2018), anterior duplication has been observed after *roboA* RNAi (Cebria and Newmark, 2007), and lateral duplication has been observed after *wnt5* RNAi (Gurley et al., 2010). The *map3k1* RNAi phenotype differs from these other patterning phenotypes, in that it involves greater disorganization, small clusters of pharyngeal cells, and even single pharyngeal cells. This scenario bears similarity to the phenotype for the eye: disorganized ectopic tissue differentiation including in small clusters and single cells. These scenarios suggest defects in the regulation of progenitor targeting for differentiation in precise locations.

### The targeting and maintenance of tissues after *map3k1* RNAi

Post-mitotic progenitors normally migrate to their target zone (TZ) and differentiate at this location. Prior work indicates that in addition to the TZ, the target tissue itself can incorporate its fate-specified progenitors and promote progenitor differentiation, even in ectopic locations (outside of the TZ) (Atabay et al., 2018; Hill and Petersen, 2018). For instance, surgical manipulations (including transplantation) that lead to ectopic eyes outside of the TZ in wild-type animals result in stable maintenance of those ectopic eyes through progenitor incorporation and differentiation as part of turnover (Atabay et al., 2018). This is enabled by the fact that progenitors in wild-type animals are specified in broad regions, giving ectopic differentiated tissues access to a constant supply of progenitors (Lapan and Reddien, 2011, 2012). Thus, at least two components appear to be capable of promoting progenitor differentiation: the TZ and the target tissue. The pharynx shows similar properties to the case of the eye – with an ectopic pharynx being maintained through progenitor incorporation and differentiation (Hill and Petersen, 2018). A surgically transplanted ectopic organ (outside of its TZ), however, will not regenerate upon its removal. Because the original TZ location of the tissue is unchanged, progenitors will target the correct location after resection of an ectopic organ (Atabay et al., 2018; Hill and Petersen, 2018).

**Figure 4.**
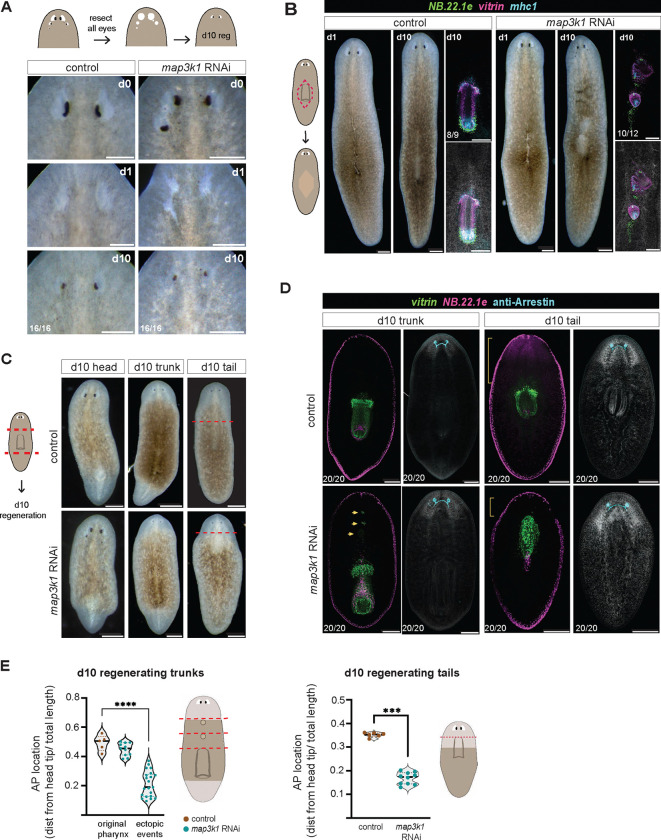
*map3k1* RNAi animals display tissue-specific regeneration at target zones. **A.** Top, schematic depicts experimental design. Bottom panels show live image examples of control and *map3k1* RNAi animal eye resections at d0, d1, and d10 post eye resection. Scale bar, 200μm. **B.** Diagram, pharynx resection. Control and *map3k1* RNAi animals at d1 and d10 post resection; accompanied by FISH of d10 pharynx regeneration using probes to *vitrin* (pharynx-specific), *NB.22.1e* (mouth and esophagus), and *mhc1* (pharynx muscle). Scale bar, 200μm**;** See also Figure 4- figure supplement 1. **C.** d10 regeneration in head, trunk, and tail fragments. An anterior-shifted pharynx regenerated in a *map3k1* RNAi tail fragment. Scale bar, 200μm**. D.** FISH images of d10 trunk and tail regenerates showing regeneration of photoreceptors (anti-Arrestin), pharynx (*vitrin*), and mouth and esophagus (*NB.22.1e*). An anterior shift in *vitrin*+ cells in d10 tail regenerate. Scale bar, 200μm. **E.** AP distributions of original trunk pharynges in control and *map3k1* RNAi animals and ectopic differentiation events in *map3k1* RNAi trunk regenerates **** p <.0001 Mann-Whitney-U test. **F.** AP distributions of regenerated pharynges in tail fragments shows an anterior shift in pharynx regeneration in *map3k1* RNAi animals compared to control animals. *** p <.0001 Mann-Whitney-U test.

Given the above reasoning, ectopic eyes in *map3k1* RNAi animals could in principle be explained by TZ movement or expansion, if *map3k1* primarily acted to control TZ locations. To assess whether TZ alteration, or some other explanation for the *map3k1* RNAi phenotype is more likely, we resected all visible eyes in *map3k1* RNAi animals to determine the location of new progenitor differentiation in the absence of a target tissue. These eye-resected *map3k1* RNAi animals grew back eyes at a comparable rate to control animals, and in the expected wild-type (normal TZ) location (Figure 4A). Notably, however, *map3k1* RNAi animals did not regenerate all other ectopic eyes that were removed. This indicates that the TZ location is maintained after *map3k1* RNAi, and that at least some progenitors are capable of migrating and differentiating at the normal TZ location in the absence of an eye. These data are consistent with a model in which *map3k1* does not primarily control the positional information read by progenitors, but instead affects the ability of progenitors to differentiate at proper locations in response to a normal positional information system – a possibility explored further below.

To determine if the pharynx target zone is also maintained in its normal, wild-type location in *map3k1* RNAi animals, we removed the entire pharynx from its normal location during progression of the *map3k1* RNAi phenotype (Figure 4B, Figure 4- figure supplement 1A). By 10 days after pharynx resection, animals regenerated a pharynx in the original pharynx position, as well as displayed an additional anterior pharynx-like structure (Figure 4B, Figure 4- figure supplement 1B). Thus, the case for the pharynx is more complex than for the eye. Regardless, these findings for the pharynx suggest that at least the normal central TZ remains and is not simply shifted anteriorly.

### The location of de novo organ regeneration after *map3k1* RNAi

To determine the location of progenitor targeting and de novo organ formation during regeneration, we amputated *map3k1* RNAi animals into head, trunk, and tail fragments and analyzed them after 10 days of regeneration. All fragments regenerated any organs that they did not already contain at the time of amputation (Figure 4C, D). Two eyes formed in approximately normal locations in *map3k1* RNAi head blastemas, rather than being posteriorly shifted or appearing in multiple locations initially. This suggests, similarly to the results from the eye resection experiments described above, that the TZ is regenerated in roughly the wild-type location in *map3k1* RNAi animals.

Prior work in the planarian species *D. japonica* showed that *map3k1* RNAi results in tail fragments regenerating pharynges in an anterior-shifted location (Hosoda et al., 2018). Consistent with this previously reported effect, pharynges regenerated more anteriorly in *map3k1* RNAi *S. mediterranea* tail fragments, just posterior to the regenerating brain (Figure 4C–E). Notably, trunks also regenerated secondary pharynx-like aggregates very close to the brain that were underdeveloped, and appeared to interfere with structures around them (Figure 4D, E). Head fragments regenerated multiple pharyngeal structures (Figure 4- figure supplement 1C). Therefore, the situation for the pharynx in regeneration is more complex than that of the eye, similar to the findings for organ resections described above.

### PCGs and pole organization are largely unaffected by *map3k1* RNAi

**Figure 5.**
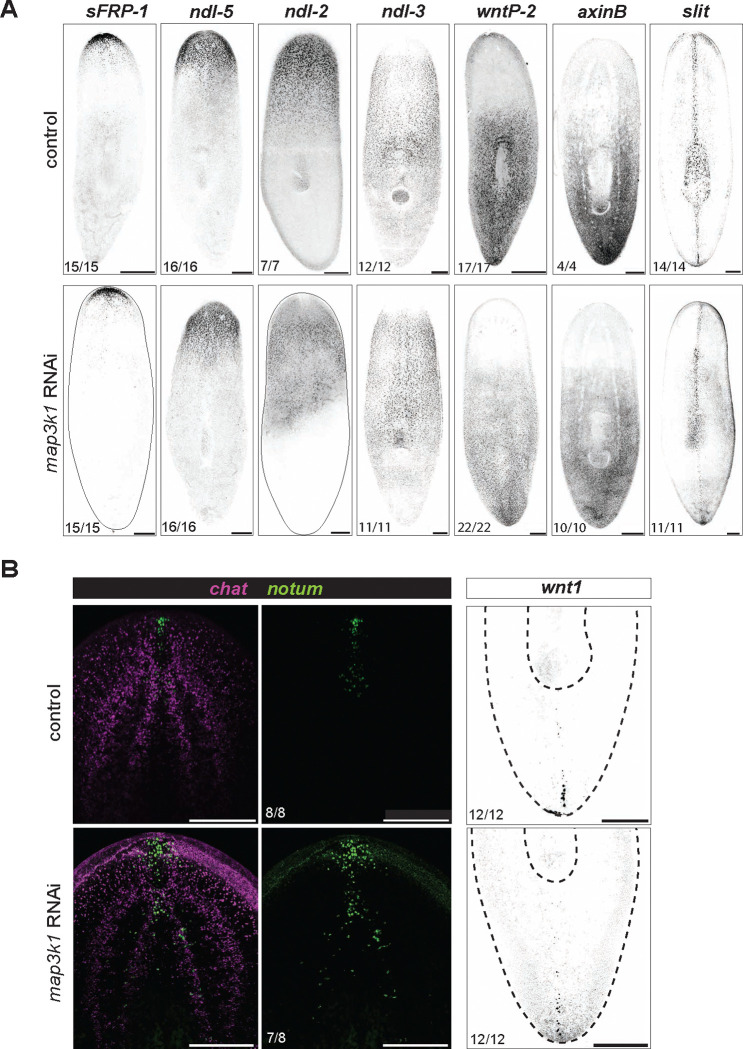
Positional information remains largely unaffected in *map3k1* RNAi animals **A.** FISH panel of position control genes (*sFRP-1, ndl2, ndl5, ndl3, wntP-2, axinB, and slit*) shows no obvious changes to positional information. Sample numbers are indicated in the lower left of each panel. Scale bar, 200μm. **B.** FISH showing some dispersion of *notum*+ cells in the heads of *map3k1* RNAi animals compared to control animals. Right panels, no obvious changes in posterior pole organization. Sample numbers are indicated in the lower left of each panel, scale bars, 200μm.

As noted above, numerous PCGs can be inhibited to cause organ duplications. However, the *map3k1* RNAi phenotype described so far is largely consistent with alteration of progenitor targeting behavior rather than global positional information shifting, as exemplified by the case of the eye. To directly examine the spatial maintenance of positional information in *map3k1* RNAi animals we labeled these animals with RNA probes for multiple PCGs. Anterior, posterior, and medial PCG expression domains were largely unaffected in *map3k1* RNAi animals – including expression domains for *sFRP-1, ndl-5, ndl-2, ndl-3, wntP-2, axinB,* and *slit* (Figure 5A, B). The *notum*+ anterior pole, which is produced from migratory *FoxD*+ progenitors (Scimone et al., 2014b), was present, but displayed some dispersal of cells, somewhat reminiscent of the eye phenotype (Figure 5B). The anterior pole also displayed some disorganization during head regeneration (Figure 4- figure supplement 1D). The posterior pole appeared normal (Figure 5B). *axinB* transcription can serves as a read-out of the posterior-to-anterior Wnt activity gradient in planarians (Iglesias et al., 2008; Reuter et al., 2015; Stückemann et al., 2017; Tewari et al., 2019). *axinB* was transcribed in a similar pattern in negative control and in *map3k1* RNAi animals, indicating that Wnt activity was maintained in a relatively normal spatial distribution (Figure 5A). These findings are consistent with results described so far: if enough progenitors can still make it to the correct TZ location, eyes will be maintained and regenerated at the normal TZ, indicating positional information still remains and defines a TZ for the eye at approximately the correct location in *map3k1* RNAi animals. The fact that ectopic eyes emerge over time in a disordered fashion suggests that it is progenitor targeting for differentiation at precise locations that is affected by *map3k1* RNAi rather than positional information.

### *map3k1* is expressed in neoblasts and migratory post-mitotic progenitors

Recent scRNA-seq work has developed annotated fate-associated clusters of planarian neoblasts and post-mitotic progenitors (King et al., 2024). If *map3k1* acts in progenitors to regulate their differentiation, is should be transcribed in these cells. Indeed, *map3k1* transcripts were present broadly across neoblast and post-mitotic progenitor clusters (Figure 5- figure supplement 1), including for the eye (Figure 5- figure supplement 1C, D), neural classes affected by *map3k1* RNAi (Figure 5- figure supplement 1E–G), and for the pharynx (Figure 5- figure supplement 1H, I). Whereas expression data alone do not necessarily *map3k1*, these data are consistent with the possibility that *map3k1* can act within migratory progenitors.

### *map3k1* is required for restricting differentiation of eye progenitors along their migratory path to the target zone

Eye and pharynx progenitors are normally specified in restricted domains (Lapan and Reddien, 2011, 2012; Adler et al., 2014; Scimone et al., 2014a; Atabay et al., 2018), (referred to here as progenitor specification zones). Within these domains, progenitors migrate to reach their target tissue at their TZ, where they differentiate (Atabay et al., 2018; Hill and Petersen, 2018). We sought to test the possibility that the ectopic differentiated cells in *map3k1* RNAi animals resulted from premature progenitor differentiation at positions on their normal migratory paths, before reaching the TZ. An alternative scenario we considered is that ectopic progenitor specification at some distant location occurs after *map3k1* RNAi and requires excessively long-range progenitor migration to reach the TZ, ultimately resulting in ectopic differentiation.

**Figure 6.**
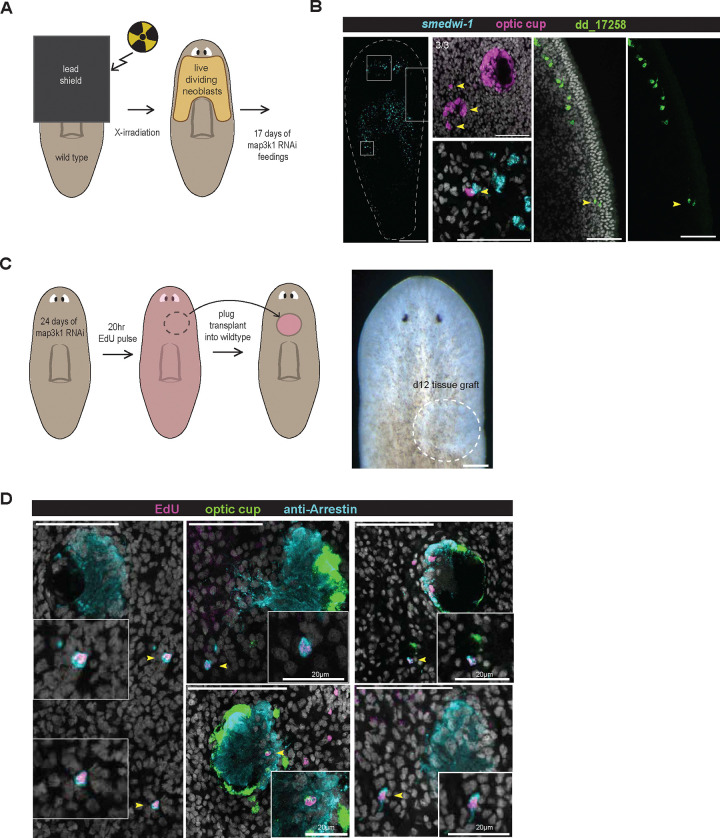
*map3k1* RNAi eye progenitors prematurely differentiate along their typical migratory path. **A.** Schematic of head-shielded irradiation experimental design. **B.** FISH showing instances of ectopic photoreceptor (anti-Arrestin), optic cup (*catalase, tyrosinase, glut3*), and dd_17258 cells born in the upper half of the animal (n=3/3 with good shielding and cells present correspond to area of leftover neoblasts). **C.** Schematic of EdU-labeled transplant experimental design. **D.** FISH and antibody staining showing examples of EdU+ eye cells from *map3k1* RNAi animals ectopically differentiated in wild-type animals (n=7/18 with EdU+ ectopic cells; n=16/18 exhibiting any ectopic eye cells outside the transplant area). Scale bar, 50μm.

Planarian neoblasts can be killed with irradiation (Bardeen and Baetjer, 1904; Reddien et al., 2005). Lead shielding can be utilized in X-irradiation experiments to locally protect neoblasts, resulting in neoblasts present only in a restricted field (Dubois, 1948; Guedelhoefer and Sánchez Alvarado, 2012; Abnave et al., 2017). Neoblasts expand only very slowly from these restricted regions (barring amputation) (Saló and Baguñà, 1985), but their postmitotic descendant cells serving as progenitors can readily migrate out from this region to differentiate at target tissues (Guedelhoefer and Sánchez Alvarado, 2012; Abnave et al., 2017; Park et al., 2023). We utilized lead shielding over the head and pre-pharyngeal region and X-irradiation to preserve neoblasts only in the anterior region where eye progenitors are primarily specified (Figure 6A). 48 hours after irradiation, we initiated RNAi of *map3k1* to observe the behavior of any eye progenitors restricted to be born within their primary eye progenitor specification zone (the zone of surviving neoblasts). 17 days after the initiation of *map3k1* RNAi, ectopic eye cells and ectopic dd_17258+ neurons were apparent (Figure 6B, C). This indicates that ectopic differentiation can occur after *map3k1* RNAi even from neoblasts restricted to undergo fate specification close to their target tissue.

Second, we EdU-labeled neoblasts in *map3K1* RNAi animals after 24 days of RNAi, then transplanted a pre-pharyngeal tissue fragment from these animals into the prepharyngeal region of unlabeled control host animals (hosts lack *map3K1* RNAi) (Figure 6D). Animals were then fixed 12 days later. This experimental design allowed assessment of the behavior of *map3k1* RNAi progenitors that migrate into a non-RNAi host environment, from within the normal eye progenitor specification zone. EdU-labeled cells migrated out of the transplantation region and differentiated into eye cells at both the normal eye location (at the TZ) and at ectopic locations in the host environment (Figure 6E). Additional ectopic eye cells were observed following transplantation that were not EdU-labeled, likely the result of additional mistargeting of *map3k1* RNAi progenitors from the transplant, but lacking EdU because of incomplete EdU labeling or from cells born after the EdU pulse (Supplemental Figure 5A, B). These observations indicate that eye progenitors originating from the normal specification zone can erroneously differentiate before reaching their target location.

### Differentiated cells in the wrong organ of *map3k1* RNAi animals

**Figure 7.**
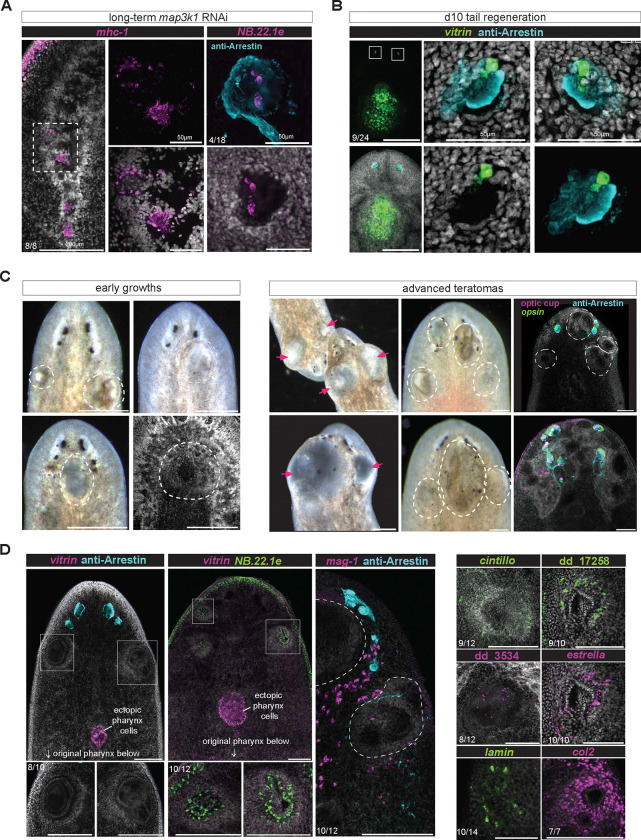
*map3k1* RNAi results in differentiation in incorrect organs and teratoma formation. **A.** FISH of long term (>6 weeks) *map3k1* RNAi animal showing clusters of *mhc1* cells that appear to have formed small structures within the lobes of the brain and ventral cords. Right panels depict *NB.22.1e* cells incorporated into the eye (anti-Arrestin). Number of animals analyzed is indicated in the lower left of each panel; Left panel scale bar, 200μm; all other panels scale bars, 50μm. **B.** FISH showing *vitrin*+ cells incorporated into the eyes of a d10 tail regenerate. n=9/24 animals contained at least one *vitrin*+ cell in one eye; left panels scale bar, 200μm. All other scale bars, 50μm. **C.** Left panel contains live images of early developing growths in *map3k1* RNAi animals, accompanied by a DAPI image of an outgrowth in between the brain lobes. Scale bars 200μm; The right panel contains live images and FISH examples of late-stage teratomas from *map3k1* RNAi animals between 12 and 16 feedings, accompanied by FISH examples showing eyes dispersed around the teratomas. Scale bars, 200μm. **D.** Left panels are FISH images showing an exclusion of *vitrin*+ cells from lateral outgrowths but a common presence of *NB.22.1e*+ cells in outgrowths. *mag1*, a parenchymal marker with transcripts abundant in cells in the neck region is also mostly excluded from outgrowths. Right panel shows examples of cell types commonly found in outgrowths: *cintillo* (neuron), dd_17258 (neuron), dd_3534 (neuron)*, estrella* (glia), anti-Arrestin (photoreceptor projections), *NB.22.1e*, *lamin* (mouth, epidermis), and *col2* (muscle). Scale bar 100μm.

We administered *map3k1* dsRNA for RNAi and let animals undergo normal long-term tissue turnover to observe the consequences of errors in progenitor targeting. We observed differentiated pharynx muscle cells within the cephalic ganglia (Figure 7A). We also observed other differentiated pharynx and epidermal cell types within the eye (Figure 7B), including in regenerated animals (Supplemental figure 5C). These striking cell-organization defects were not previously observed in the patterning phenotypes observed following RNAi of PCGs. We suggest that this defect highlights the risk to tissue architecture of not enacting tight spatial regulation of differentiation, especially in a biological context where progenitors are spatially dispersed and migratory. Ectopic differentiated cells can become incorporated into inappropriate tissue environments where they would normally never be observed, potentially disrupting tissue structure and function.

### *map3k1* RNAi animals develop teratomas

Long-term *map3k1* RNAi in animals undergoing tissue turnover without injury led to the inevitable emergence of tissue growths (Figure 7C, D, Figure 6- figure supplement 1C). These growths predominantly formed in the anterior of the animal and presented as a heterogeneous collection of cell and tissue types. *vitrin*+ cells and gland cells (*mag1*) were not as common in lateral outgrowths. Cell types that were present in these teratomas included eye cells, various neuron types from the central nervous system (*cintillo*+, dd_17258+, dd_3524+), glia, muscle cells, and *NB.22.1e* cells (Figure 7D). A similar patterning defect has been observed in planarians with a defect in progenitor migration caused by *integrin* RNAi (Bonar and Petersen, 2017; Seebeck et al., 2017). It is known that differentiated tissues, such as the eye, can trap their own progenitors and lead to their differentiation (Atabay et al., 2018; Hill and Petersen, 2018). This suggests that ectopic differentiation in inappropriate locations in *map3k1* RNAi animals can trap more progenitors and lead to an inappropriate aggregate of differentiated cells with aberrant pattern and organization. This highlights a further risk to the formation of tissue architecture if the differentiation of migratory progenitors is not tightly controlled spatially.

## Discussion

**Figure 8.**
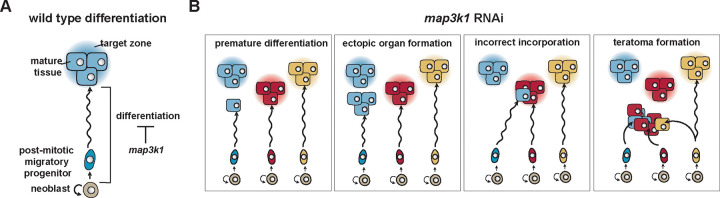
Model: Map3K1 restricts migratory progenitor differentiation until they reach their target. **A.** Schematic showing the inhibition of differentiation of a specific progenitor until reaching its target tissue at the target zone. **B.** Defects observed with *map3k1* RNAi, highlighting significance of restricting differentiation until suitable contextual cues are reached.

Planarians display continuous turnover of adult tissues through the fate specification and differentiation of adult stem cells called neoblasts (Reddien, 2018). Fate specification in neoblasts can occur regionally (such as in the head for eyes), but is still spatially broad and intermingled. Fate-specified neoblasts (specialized neoblasts) produce progeny cells (post-mitotic progenitors) that migrate to precise locations for local, highly patterned differentiation. Progenitor targeting for local differentiation requires regulation from stem-cell-extrinsic signals, in the form of regionally expressed genes in muscle that comprise adult planarian positional information. How positional information interfaces with neoblasts and post-mitotic progenitors at the molecular level to regulate migratory assortment and differentiation only at precise locations is unknown and a fundamental problem of planarian regeneration and progenitor differentiation regulation. We sought factors that might be required for this process through RNAi-based gene perturbation experiments, and uncovered a requisite role in the spatial regulation of stem cell differentiation for the *map3k1* gene, which encodes an ortholog of vertebrate MAP3K1*. map3k1* RNAi animals displayed an abnormal pattern of progenitor differentiation, with differentiation occurring in disorganized locations along post-mitotic progenitor migratory paths. We suggest a model in which *map3k1* is required for the spatial restriction of differentiation in stem cell lineages until suitable differentiation cues are encountered, either in the form of positional information (a target zone) or cues from target tissues (Figure 8A).

The defect in spatial restriction of progenitor differentiation following *map3k1* RNAi can lead to dramatic tissue patterning defects, including the differentiation of ectopic isolated cells that normally only appear in groups (e.g., isolated photoreceptor neurons), the emergence of ectopic organs, differentiated cells from one tissue type being present within an incorrect organ, and teratoma formation (Figure 8B). These tissue mispatterning attributes highlight the significance of spatial regulation of differentiation during targeting of migratory progenitors for maintaining and regenerating adult pattern. This process might prove important development in many organisms, and be particularly important in the context of adult regeneration, where tissue scale can be large and adult progenitors at least in some contexts can be challenged to migrate large distances before differentiating.

There is some differentiation of eye progenitors in the tail of *map3k1* RNAi planarians, far from the normal location of eye progenitor production and differentiation. However, only a very low rate of eye progenitor production was observed in the tail of *map3k1* RNAi animals, and a comparable low frequency of eye-specialized neoblasts were present in control tails. One interesting possibility that can be investigated in the future is that there is some noise in the spatial regulation of stem cell fate specification in planarians that does not normally contribute to errors in the spatial distribution of differentiated cells because of prevention of differentiation until cells reach their appropriate location. The progenitor specification domain for pharynx cells, by contrast, appeared to expand anteriorly after *map3k1* RNAi, indicating a possible role in the spatial regulation of progenitor specification in some cases.

Several observations suggest that the defects in targeting in *map3k1* RNAi animals involves a process within the neoblasts and their post-mitotic progenitor descendants, rather than in a role for *map3k1* in establishing the progenitor-extrinsic cues (e.g., muscle PCGs) that facilitate progenitor targeting. First, PCG expression patterns themselves were largely normal following *map3k1* RNAi. Second, ectopic differentiation after *map3k1* RNAi was more spatially disorganized than is observed for patterning phenotypes that occur following the shifting of PCG coordinates (such as a posterior line of ectopic eyes in *ndk* RNAi animals (Cebria et al., 2002) or a lateral line of ectopic eyes in *wnt5* RNAi animals (Atabay et al., 2018)). Third, ectopic cells following *map3k1* RNAi were frequently isolated, as opposed to appearing exclusively in organized aggregates or forming ectopically placed organs. Fourth, transplantation of EdU-labeled tissue grafts from *map3k1* RNAi animals into the pre-pharyngeal region of control animals showed instances of ectopic differentiation in wild-type host tissue. Finally, during regeneration or following eye resection, progenitors can still be targeted to the correct target zone for the eye, indicating that the eye target zone still remains after *map3k1* RNAi. One possibility is that Map3k1 applies a brake on differentiation in progenitors that is released or overcome by some cue associated with a progenitor reaching its target zone or by interacting with its target tissue.

Map kinase signaling cascades are versatile pathway modules found throughout the animal kingdom and are involved in diverse processes (Widmann et al., 1999). The kinases and accompanying cofactors in these modules enable the regulated activation of various Map kinases (e.g., ERK1/2, p38, JNK) (Suddason and Gallagher, 2015). Map kinase kinase kinases (MAP3Ks) are one of the first activated proteins in these cascades, often responding to receptor tyrosine kinase signaling at the cell membrane. In mammals, there are 24 characterized MAP3Ks: MAP3K1 through MAP3K21, B-Raf, C-Raf, and A Raf, which activate downstream MAP2K proteins through phosphorylation. Among the MAP3K proteins, MAP3K1 is unique in its domain composition. In particular, MAP3K1 orthologs are the only MAP3K with a PHD domain (Suddason and Gallagher, 2015). The MAP3K1 domain architecture enables a role in ubiquitination as well as kinase activity (Pham et al., 2013). In mammals, *map3k1* has a role in regulating many processes such as proliferation, differentiation, division, and cell death (Suddason and Gallagher, 2015). *map3k1* gene functions are not well characterized in most invertebrate systems, and *Drosophila* and *C. elegans* have no identified *map3k1* orthologs (Widmann et al., 1999). Planarians therefore present an attractive invertebrate model for dissection of *map3k1* function. There is evidence for the role of various Map-kinases (e.g., ERK, MEK, RAS, p38) in planarian regeneration, particularly in the blastema formation and the wound and missing tissue response programs (Tasaki et al., 2011a; Tasaki et al., 2011b; Owlarn et al., 2017; Wang et al., 2020). *map3k1* has been studied in a different planarian species, *D. japonica* (Hosoda et al., 2018). This work suggested a possible role for *map3k1* in the scaling and patterning of the trunk and head regions of regenerating animals, proposed to be through alternation of ERK and β-catenin gradients (Hosoda et al., 2018). Additionally, *map3k1* has been implicated in germ cell proliferation and terminal differentiation of stem cells in the parasitic flatworm *E*. *multilocularis* through JNK signaling (Stoll et al., 2021). It will be of interest to further dissect the molecular role of *map3k1* in planarian progenitor differentiation, and to determine whether *map3k1* orthologs have similar roles in regulating differentiation in additional regenerative organisms.

Adult patterning systems in some organisms can rely on a spatially coarse progenitor specification system, migratory assortment of progenitors, and local differentiation. This process involves progenitor transitions from spatially broad and disorganized, to local and highly patterned as cells transit from stem cell to post-mitotic migratory progenitor. This suggests that regulation of terminal differentiation must have a central role in pattern production. Progenitor-extrinsic positional information has a prominent regulatory role in this process, and some mechanism(s) could exist within the progenitors themselves to enable spatial restriction of differentiation until suitable cues have been received. We suggest that *map3k1* acts within planarian progenitors to mediate such spatial restriction on differentiation, and that this is critical for preventing mistargeting differentiation to the incorrect location or organ, and to prevent teratoma formation.

## Materials and methods

### Animal husbandry and surgery

Asexual *S. mediterranea* clonal strain CIW4 was used for all experiments. Animals were cultured in static 1x Montjuic water (1.6 mmol/l NaCl, 1.0 mmol/l CaCl_2_, 1.0 mmol/l MgSO_4_, 0.1 mmol/l MgCl_2_, 0.1 mmol/l KCl and 1.2 mmol/l NaHCO_3_ prepared in Milli-Q water) at 20°C. Amputations were performed under cold conditions (~4C) with a scalpel. Animals were fed homogenized beef liver weekly, with water changed biweekly. Animals were starved for approximately seven days before experiments.

### Whole-mount fluorescent in situ hybridization (FISH)

Animal mucus was removed using 5% N-acetylcysteine in PBS; animals were then fixed with 4% formaldehyde in PBST for 20 minutes, with rocking. Animals were then washed with PBST, incubated in 1:1 PBST:methanol, then stored in 100% methanol at −20C until ready for bleaching. Animals were moved into mesh baskets in a 24-well plate where all remaining steps were carried out. Animals were placed on a light source to bleach for 1.5 hours in a bleaching solution (5% formamide, 0.5x SSC, and 1.2% hydrogen peroxide). After 2 PBST washes, animals were then treated with 5 mg/ml Proteinase K for 10 minutes, followed by 4% formaldehyde post-fixation in PBST.

RNA probes were transcribed using DIG, FITC, or DNP-modified nucleotides, allowing signal amplification through the use of DIG, FITC, and DNP antibodies conjugated to an HRP. Probes were diluted in Hybe solution (1:800) (50% deionized formamide, 5x SSC, 1 mg/mL yeast RNA, 1% Tween-20, 5% dextran sulfate), and left to incubate overnight. The following days, we performed antibody incubations at 4C overnight using anti-DIG-POD (1:1500, Roche; 10% western blocking solution (Roche) ), anti-FITC-POD (1:2,000, Roche; 5% horse serum, 5% western blocking solution), and anti-DNP-HRP (1:100, Perkin-Elmer; blocking solution with 10% inactivated Horse Serum). Tyramide signal amplification involved incubating in rhodamine (1:1,000), fluorescein (1:1,500), or Cy5 (1:300) in borate buffer (0.1M boric acid, 2M NaCl, pH 8.5) containing 0.0003% hydrogen peroxide for 10 minutes. Samples were incubated in 1% sodium azide for 2 hours to inactivate the HRP. Blocking and antibody incubations then occurred for detection of the second probe. Animals were incubated overnight in 1mg/mL DAPI solution at 4C. Animals were mounted on coverslips in ProLong Gold Antifade Mountant (Thermo Fisher).

### EdU labeling and detection

F-ara-EdU (Click Chemistry Tools) was diluted in Dimethyl sulfoxide (DMSO) to 200mg/ml, then diluted in static 1x Montjuic water to 1.25mg/mL. Animals were split into 10 animals per well in a 12-well plate, then soaked in 1.25mg/mL EdU solution for 20 hours following 1 week of starvation. EdU solution was replaced with 5mg/mL Instant Ocean sea salt dissolved in Milli-Q water. Prior to probe hybridization in the *in situ* hybridization protocol, following proteinase K and 4% formaldehyde incubations, cells were incubated in a “click reaction”- 1% 100 mM CuSO4, 0.1% 10 mM TAMRA-Azide-fluor 545 (Sigma-Aldrich), and 20% 50 mM ascorbic acid in PBS- for 30 minutes in the dark, proceeded by 6 PBST washes and continuation of the probe hybridization step.

### RNA interference

dsRNA for RNAi was prepared using in vitro transcription with T7 polymerase (Promega). Template was generated with PCR; primers contained T7 promoters. RNA was resuspended in water and dsRNA annealed. *C. elegans unc-22* dsRNA was used as the negative control for all RNAi experiments.

### Transplantation

EdU plug transplants were performed using *map3k1* RNAi animals, 12 hours following a 20 hour EdU pulse, as the donor to a recipient wild type animal. Donor animals were anesthetized with 0.2% chlorotone solution, followed by an incubation in Holfreter’s solution, then placed on an ice block covered in Whatman filter paper moistened with 1x Montjuic water to surgically manipulate with a clean scalpel. EdU+ *map3k1* RNAi donor animals and wild type recipients both had a center portion of their pre-pharyngeal regions removed. The pre-pharyngeal donor graft from the *map3k1* RNAi animal was placed in the EdU− wild type recipient’s pre-pharyngeal region. Recipients were then gently covered with cigarette paper soaked in chilled Holfreter’s solution and transferred to a small petri dish with just enough Holfreter’s solution to cover the bottom of the dish. Animals were put at 10C for 20 hours; the following day, transplant recipients were gently recovered and put into 1x Montjuic water containing .1% gentamicin (Gibco).

### Shielded irradiation

Animals were irradiated using a Gammacell-40 137-cesium source. For shielded irradiation experiments, animals were anesthetized with 0.2% chlorotone, then arranged on Whatman filter paper in a petri dish sitting on ice. Animals were oriented to have their anterior half covered by the lead shield placed over the petri dish. Samples were placed in a Gammacell chamber and exposed to 3000 Rad of unidirectional X-irradiation. Animals were rescued with 1x Montjuic water and stored in 1x Montjuic water with .1% gentamicin (Gibco) until further experimentation.

### Image analysis and statistical analysis

For cell counts, the AP axis was binned into 6 regions according to anatomical landmarks. AP_1 (head tip to bottom of brain), AP_2 (bottom of brain to top of pharynx), AP_3 (top of pharynx to middle of pharynx), AP_4 (middle of pharynx to bottom of pharynx), AP_5 (top half of the tail) and AP_6 (bottom half of the tail). Each animal had one data point for each of the 6 bins. Graphpad Prism software was used for graph generation and statistical analysis using a Mann-Whitney-U test with a significance cut off of p=.05. For cell count comparisons in the tail, Graphpad Prism software was used for graph generation and statistical analysis using a student t-test with a significance cut off of p=.05.

## Figures and Tables

**Figure 1- figure supplement 1.**
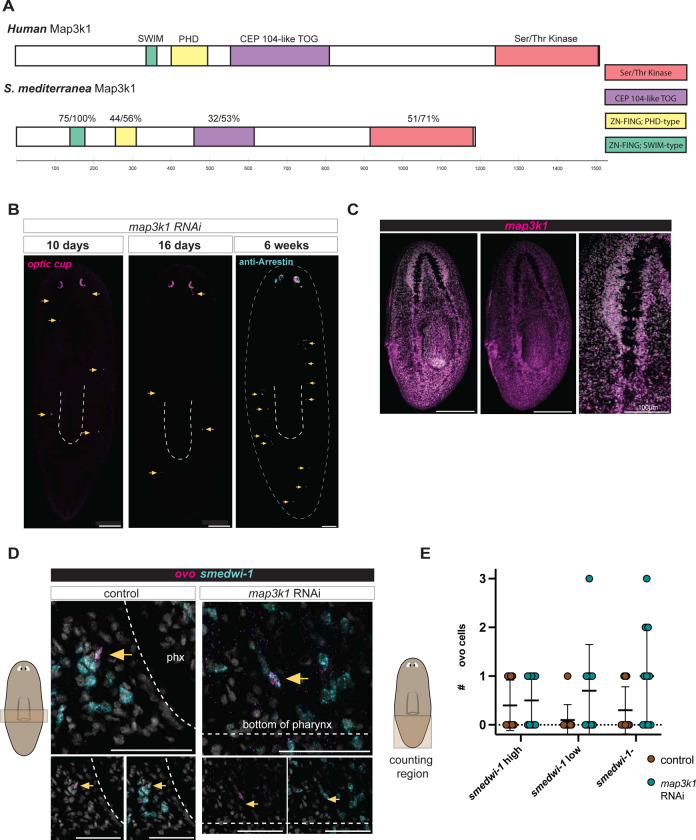
*map3k1* RNAi results in differentiated eye cells throughout the AP axis, and no overt change in eye progenitor distribution. **A.** Domain structure of human and *Schmidtea mediterranea* MAP3K1. % identity/ similarity is displayed above each domain. **B.** FISH of *map3k1* RNAi animals at 10 days, 16 days, and 6 weeks of RNAi shows optic cup (*catalase1, tyrosinase, glut3*) in the posterior at early timepoints. **C.** FISH with an RNA probe to the *map3k1* gene in a wild-type animal. Scale bar, 200μm for full body images; Scale bar, 100μm for closeup of the brain. **D.** FISH showing double-positive *ovo*+ ; *smedwi-1*+ cells near the posterior half of the pharynx. Scale bars, 50μm. **E.** quantification of *smedwi-1* high, *smedwi-1* low, and *smedwi-1* negative *ovo*+ cells in the tails of *map3k1* RNAi and control animals. No significant change is observed between the number of *smedwi-1*+ ; *ovo*+ cells in the tail between *map3k1* RNAi and control animals.

**Figure 2- figure supplement 1.**
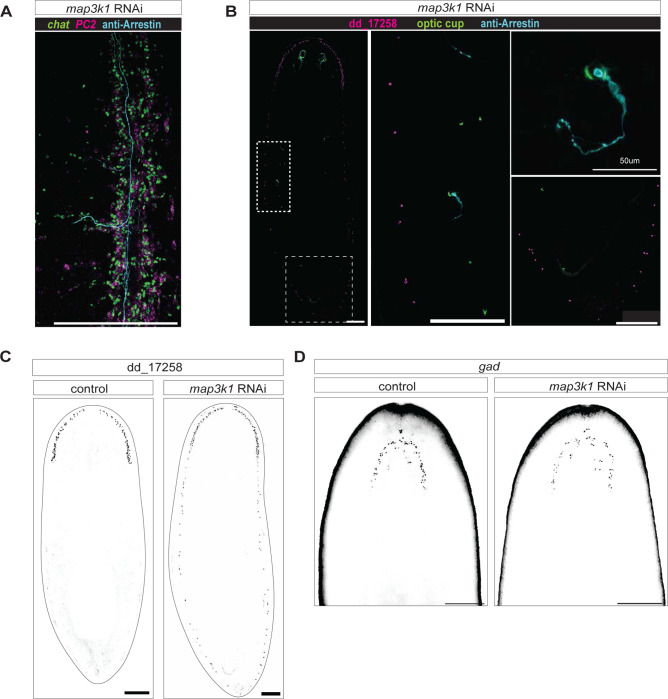
*map3k1* RNAi results ectopic brain branches posterior to the brain and posterior expansion of some neural cell types. **A.** Close-up FISH of ventral nerve cord (VNC) and branches in a *map3k1* RNAi animal. Photoreceptor projections (anti-Arrestin) are travel along the VNC and into ectopic branches labeled by the pan-neural markers *chat* and *pc2*. Scale bar, 100μm. **B.** FISH of dd_17258+ cells and optic cup cells (*catalase1, tyrosinase, glut3*), together with antibody labeling of photoreceptors (anti-Arrestin), extending down the AP axis. All scale bars, 200μm, unless otherwise specified in the lower left. **C.** FISH showing expansion of dd_17258 cells down the entire AP axis in a *map3k1* RNAi animal. Scale bar, 200μm. **D.** FISH showing an unchanged neural cell type (*gad*) in *map3k1* RNAi and control animals. Scale bar, 200μm.

**Figure 4- figure supplement 1.**
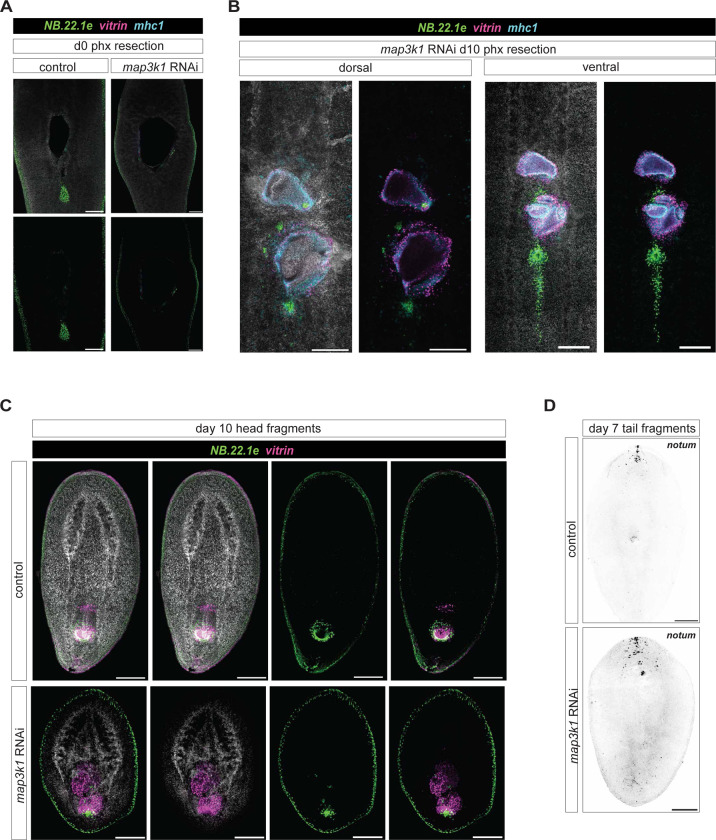
*map3k1* RNAi animals can undergo tissue-specific and whole-body regeneration, with some errors in pharynx organization. **A.** FISH following pharynx resection at day zero (d0) with the markers *vitrin* (pharynx-specific), *NB.22.1e* (mouth and esophagus), and *mhc1* (pharynx muscle). Scale bars, 200μm. **B.** Example of *map3k1* RNAi d10 pharynx resection. FISH shows dorsal (left) and ventral (right) views of the same animal, showing disorganized pharynx regeneration in *map3k1* RNAi animals. **C.** FISH of d10 head regeneration showing disorganized regeneration of the pharynx using *vitrin* and *NB.22.1e* RNA probes. Scale bars, 200μm. n=10/10. **D.** FISH showing dispersed *notum*+ cells in a d7 tail regenerate compared to control animals. Scale bar, 200μm

**Figure 5- figure supplement 1.**
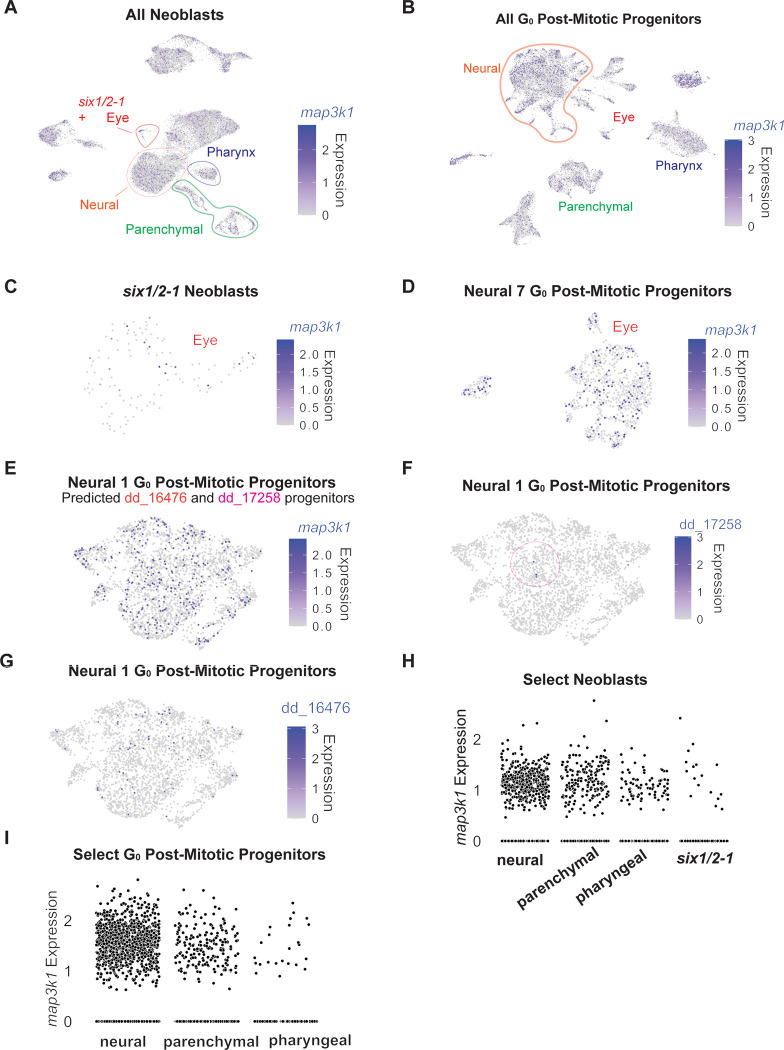
*map3k1 i*s expressed in neoblasts and post-mitotic progenitors. **A.** *map3k1* is expressed broadly across neoblast clusters. Plot points are ordered. **B.** *map3k1* is expressed broadly across post-mitotic progenitors; plot points are ordered. **C.** *map3k1* is expressed in some *six-1/2*+ neoblasts, which contain eye neoblasts; plot points are ordered. **D.** *map3k1* is expressed in some G0 post-mitotic eye progenitors; plot points are ordered. **E.** *map3k1* is expressed within the predicted progenitor cluster (Neural 1) of the affected cell types: dd_17258 and dd_16476; plot points are ordered. **F.** dd_17258 mature marker expression within its predicted progenitor cluster (Neural 1); plot points are ordered. **G.** dd_16476 mature marker expression within its predicted progenitor cluster (Neural 1); plot points are ordered. **H.** *map3k1* expression in neural, parenchymal, pharyngeal, and *six1/2*+ neoblast clusters. **I.** *map3k1* expression in neural, parenchymal, and pharyngeal post-mitotic progenitor clusters.

**Figure 6- figure supplement 1.**
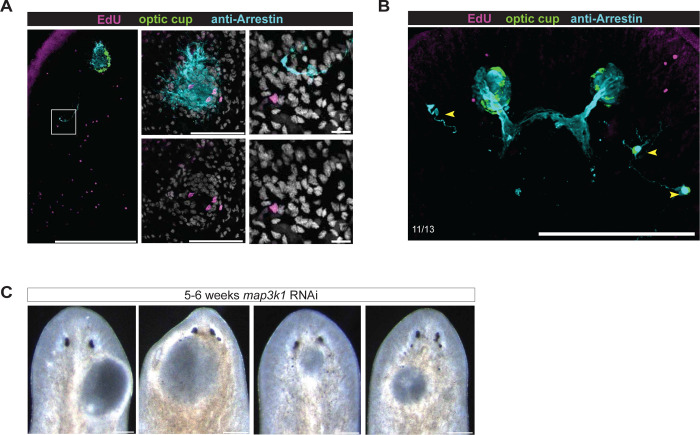
*map3k1* RNAi animals display to improper progenitor targeting and teratoma formation. **A.** FISH showing EdU+ photoreceptors (anti-Arrestin) in the eye of the recipient animal. These cells differentiated from *map3k1* RNAi progenitors born during the EdU pulse of the donor animal, following wild-type migratory cues out of the graft and into the recipient eye. Scale bar, 200μm. **B.** FISH image of EdU− ectopic eye cells in recipient wild-type animals after EdU plug transplantation. Scale bar, 200μm. **C.** Live images of early growths that will eventually become teratomas in *map3k1* RNAi animals. Scale bar, 100μm.
